# Up-Conversion Emissions from HfO_2_: Er^3+^, Yb^3+^ Nanoparticles Synthesized by the Hydrothermal Method

**DOI:** 10.1021/acsomega.4c00808

**Published:** 2024-08-28

**Authors:** Marlen Deyanira Méndez-Castillo, Manuel García-Hipólito, Luis Zamora-Peredo, Adriana Sumoza-Toledo, Irma Yadira Izaguirre-Hernández, Rocio Guadalupe Casañas Pimentel, Jaime Martínez-Castillo, Leandro García-González, Julián Hernández Torres, Adriana Báez-Rodríguez, Ciro Falcony

**Affiliations:** † Centro de Investigación en Micro y Nanotecnología, Universidad Veracruzana, Calzada Adolfo Ruiz Cortines 455, Fraccionamiento Costa Verde, Boca del Río, Veracruz 94292, Mexico; ‡ Instituto de Investigaciones en Materiales, Universidad Nacional Autónoma de México, Circuito Exterior, Ciudad Universitaria, Coyoacán, CDMX 04510, Mexico; § Universidad Veracruzana, Instituto de Investigaciones Medico - Biológicas, Veracruz 94292, Mexico; ∥ Centro de Investigación de Ciencia Aplicada y Tecnología Avanzada, Instituto Politécnico Nacional, CDMX 04510, Mexico; ⊥ Centro de Investigación y de Estudios Avanzados del Instituto Politécnico Nacional, A.P. 14-740, CDMX 07360, Mexico

## Abstract

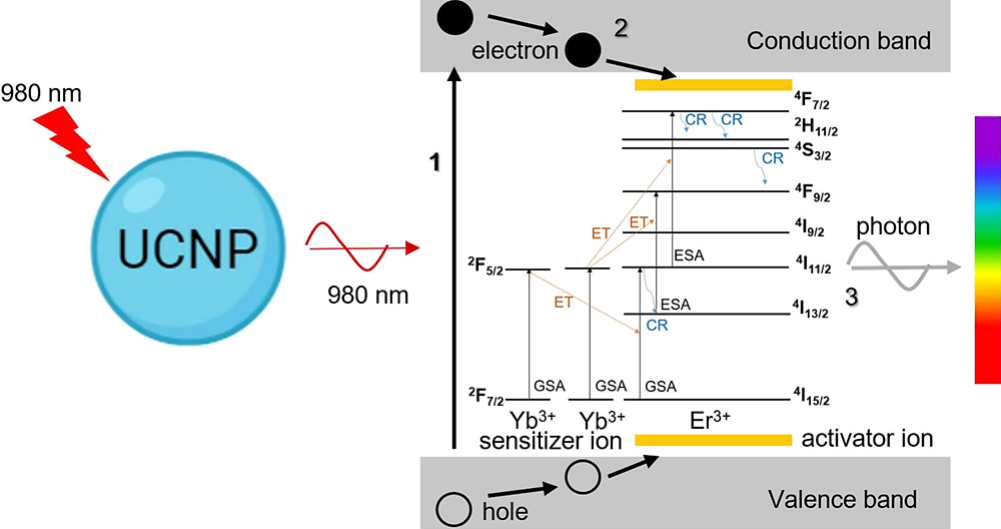

Up-conversion emission from HfO_2_ nanoparticles, as a host lattice, doped with Er^3+^ and Yb^3+^ ions and codoped with alkaline cations Li^+^ and Na^+^ obtained. The HfO_2_ nanoparticles, about 80 nm in diameter, were synthesized by the hydrothermal method at 200 °C for 1.3 h, and an additional heat treatment at 1000 °C was necessary to ensure the dopants incorporation into the host lattice. These nanoparticles were studied by means of XRD, Raman Spectroscopy, SEM, EDS, PL, CL, and up-conversion luminescence. First, the doping was performed with Er^3+^ ions in different percentages. The photoluminescence and cathodoluminescence studies showed an inefficient emission, and only at 7 at % Er^3+^ ions, the sample presented emissions at 522, 545, and 656 nm corresponding to the transitions of the Er^3+^ ions. So, codoping was carried out, and HfO_2_: Er^3+^/Yb^3+^ generated an efficient conversion process. The atom percentage of Yb^3+^ ions was fixed (7 at % Yb^3+^), and the Er^3+^ content was varied, showing the highest emission intensity at 3 at % Er^3+^ ions. Subsequently, the up-conversion emission intensity was optimized by varying the percentage of Yb^3+^ ions and keeping the Er^3+^ ion content fixed (3 at %). Adding cations such as Na^+^ and Li^+^ in different percentages, a notable improvement of the up-conversion emission intensities in the HfO_2_: Er^3+^/Yb^3+^ nanoparticles was obtained. The up-conversion emission bands observed were located at ∼523 and 544 nm, corresponding to the electronic transitions ^2^H_11/2_ → ^4^I_15/2_ and ^4^S_3/2_ → ^4^I_15/2_, respectively. While the bands at ∼652 and 673 nm correspond to the transition ^4^F_9/2_ → ^4^I_15/2_, respectively. The excitation of these materials with infrared radiation (980 nm) produced noticeable emission bands in the red spectral range, whereas excitation with accelerated electrons (CL) generated prominent bands in the green region.

## Introduction

Study of luminescent nanomaterials has been growing exponentially, thanks to the multiple applications they have from smart displays,^1^ scintillators,^2^ CMOS devices,^3^ light-emitting diodes,^4−6^ fluorescent lamps,^7^ sensors,^8^ optoelectronic devices,^9^ and solar cells^10,11^ to medical and/or biological applications such as theragnostic or bioimaging.^12,13^ Luminescent nanomaterials present energy emission phenomena called Stokes or anti-Stokes shifts. The first one presents an emission wavelength longer than the exciting radiation, which is commonly on the order of UV (ultraviolet), and this process is also known as down conversion (DC). In the second one, the opposite process occurs, the emission wavelength is shorter than the excited radiation one, which, in this case, is in the IR (infrared) range, and this effect is known as up-conversion (UC).^4^ To obtain each of these phenomena, a host lattice and inserted activator ions are needed. The inorganic crystalline (or amorphous) host can be a fluoride, sulfide, or oxide compound.^14−21^ Metal oxides are known for their good physical and chemical stability, low phonon frequency, and large energy gap, to mention some of their most important characteristics.^21,22^

HfO_2_ (hafnium oxide) was tested as an alternative for silica substitution in the semiconductor industry as well as in other uses in nanoelectronic devices.^23−30^ The characteristics that it possesses are its wide ban gap (5.68 eV),^2^ a high refractive index,^8^ transparency in the visible range,^31,32^ great dielectric constant,^7^ high melting temperature (2774 °C), good chemical stability,^33^ high crystallographic density, and so forth,^3,34^ making it suitable for optical applications.^35^ Among its various applications in engineering,^8,31^ it is found as an ideal candidate for the creation of luminescent materials where it plays the role of host lattice. Recent studies carried out with HfO_2_ report its low toxicity and a good response in nanomedicine applications such as injectable nanocolloids and nanotheragnostic materials.^36−39^ There are different works that report the use of HfO_2_ as a host lattice doped with Ba,^8^ Er^3+^,^25^ Ce^3+^,^31^ Tb^3+^,^34^ Eu^3+^,^40^ Au,^41^ Al,^42^ Cu,^43^ Mn^2+^,^44^ Gd,^45^ Ti,^46^ La, and^47^ Sm^3+^^48^ and codoped such as Er^3+^/Yb^3+^,^7^ Ce^3+^/Dy^3+^,^49^ Lu^3+^/Eu^3+^,^50^ Er^3+^/Yb^3+^/Li^+^,^51^ and La/Er^3+^/Yb^3+^/Li^+^,^3^ confirming that it is a good host lattice for Ln^3+^ ions.

Luminescent nanomaterials can generate UC or DC emissions, depending on the excitation source and nature of the Ln^3+^ ions used. The UC is given by the absorption of two or more lower energy photons, producing a higher energy photon.^3,52^ The main advantage of UC is its excitation source that operates in the infrared region, which implies a lower cost and a long lifetime of the devices.^7^ Er^3+^ and Yb^3+^ ion pair is known for their good UC emission using a 980 nm excitation source. Er^3+^ ion is known for its long lifetime in the ^4^I_11/2_ state^33,51^ and emissions by green and red lights. In general, the efficiency of UC in Er^3+^ is low, and it is highly dependent on the amount of dopant ions as well as the aid of a sensitizer such as Yb^3+^. The combination of the Yb^3+^ and Er^3+^ ions as dopants in phosphors to obtain UC was reported in different matrices: NaYF4:Er^3+^/Yb^3+^,^53,54^ BaYO:Er^3+^/Yb^3+^,^55^ K_0.3_Bi_0.7_F_2.4_:Yb^3+^/Er^3+^,^56^ NaGdF_4_:Yb^3+^/Er^3+^,^57^ Sr_2_GdF_7_:Yb^3+^/Er^3+^,^58^ KNbO_3_:Yb^3+^/E^3+^,^59^ KGdF_4_:Yb^3+^/Er^3+^,^60^ BaY_2_O_4_:Yb^3+^/Er^3+^,^19^ YVO_4_:Yb^3+^/Er^3+^,^61^ Gd_2_O_3_:Yb^3+^/Er^3+^,^20^ Y_2_O_3_: Yb^3+^/Er^3+^,^21^ LaNbO_4_:Yb^3+^/Er^3+^,^62^ and ZrO_2_:Er^3+^/Yb^3+^.^4^ In the works, the good coupling between Er^3+^ and Yb^3+^ ions stood out. For example, in 2018, Masashi Hanioka et al. concluded that the UC emission with these ions is dependent on the host lattice structure and the energy transfer between them, as well as the separation distance that they have in relation to the composition of the host lattice.^52^ In other studies, it was reported that the incorporation of Li^+^ can considerably increase the UC emission, since adding this ion modifies the crystalline structure, distorting the crystalline field around the Er^3+^ ion, which means that prohibited transitions can be carried out, and with this, the UC emission can be improved.^51,63,64^ Just as Li^+^, Na^+^, and K^+^ can perform this type of effect when they are added to UC materials. However, Li^+^ stands out as the most appropriate, according to its low ionic radius. When Li^+^ ions are incorporated in the host lattice, the interatomic distance between Er^3+^ ions increases, and it reduces relaxation processes.^3^

There are scarce reports of HfO_2_ codoped with Yb^3+^/Er^3+^; in 2010, L. A. Gomez et al. studied the structural differences and UC luminescence of ZrO_2_ and HfO_2_ doped with Er^3+^ and Yb^3+^ synthesized by the sol–gel technique, under the 980 nm excitation.^7^ In 2015, Carmona Tellez et al. reported the behavior of HfO_2_ powders doped with Er^3+^/Yb^3+^/Li^+^ obtained by the simple evaporation method, and this material was embedded inside a polyester film to fabricate the UC films.^51^ In 2020, Mariscal Becerra et al. studied the structural behavior, UC, and DC luminescence of HfO_2_ doped with La^3+^ /Er^3+^ /Yb^3+^ and Li^+^ synthesized by the solvent evaporation technique, where the main objective was to study host lattice modification with the addition of La^3+^ ions.^3^

One of the limitations of this type of material is the UC efficiency. Therefore, in this work, the main objective was to obtain the optimal emission thorough the variation of concentration of dopants and codopants (HfO_2_:Er^3+^, HfO_2_: Er^3+^/Yb^3+^, HfO_2_:Er^3+^/Yb^3+^/Na^+^, and HfO_2_:Er^3+^/Yb^3+^/Li^+^) and to reach an improvement in efficiency. The red emission due to the UC process was possible to observe by the naked eye at natural light when phosphor nanoparticles were excited by the 980 nm wavelength. Also, green emissions were obtained when the material was excited by accelerated electrons.

## Experimental Details

The HfO_2_:Er^3+^, HfO_2_:Yb^3+^/Er^3+^, HfO_2_:Yb^3+^/Er^3+^/Na^+^, and HfO_2_:Yb^3+^/Er^3+^/Li^+^ UCNPs were synthesized by the hydrothermal method, using solutions 0.04 M of HfCl_4_ (Sigma-Aldrich 98% purity) and NaOH (Golden Bell 97% purity) as precipitating agent; an optimized pH = 14 was used. The dopant ion sources were YbCl_3_ ·6H_2_O (Alfa Aesar 99.9% purity), Er(OOCCH_3_)_3_ ·4H_2_O (Alfa Aesar 99.9% purity), and LiCl (Alfa Aesar 98% purity). All precursor solutions prepared using deionized water as a solvent were placed into a Teflon container inside a 30 mL capacity stainless steel autoclave and heat-treated at 200 °C for 1.3 h. The autoclave was then allowed to cool to room temperature, and, subsequently, the resulting precipitates were rinsed several times with deionized water and dried at 85 °C for approximately 3 h. Finally, the obtained nanoparticles were subjected to thermal treatment (TT) at 1000 °C for 2 h to ensure the dopant ions incorporation into the host lattice. Structural properties of the samples were characterized by X-ray diffraction (XRD) with a Bruker D8 diffractometer. Photoluminescence was evaluated by a JOBIN YVON HORIBA spectrofluorometer, and in the case of UC measurements, the samples were irradiated at 980 nm with a BWF1 Fiber Coupled Laser System. Cathodoluminescence was tested with the use of a cathode ray tube, applying a voltage of 5 kV with a current of 0.3 mA. Raman spectra were recorded by a Thermo Scientific with a 532 nm excitation laser. SEM images were obtained by a scanning electron microscope JEOL JSM-7600, and the chemical composition was determined by means of energy dispersive spectroscopy (EDS).

## Results and Discussion

### Crystallographic Properties

**Figure 1:**
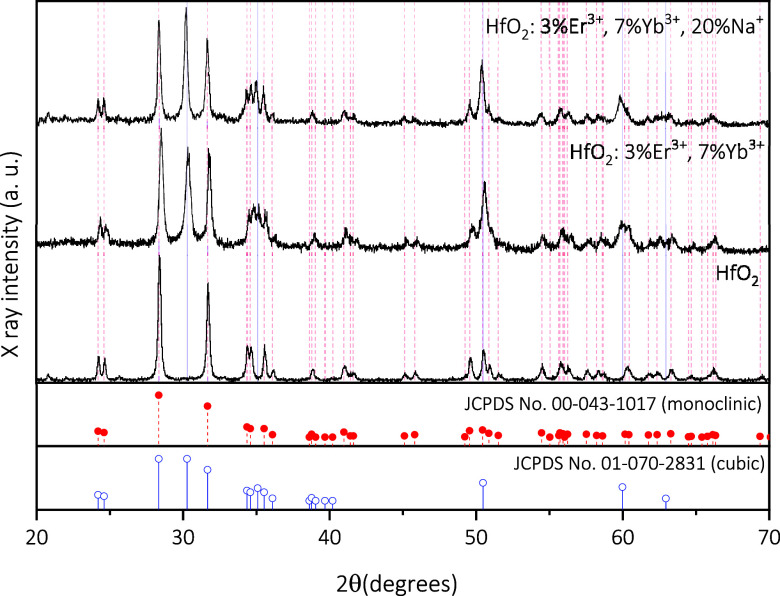
XRD diffractograms of HfO_2_, HfO_2_: Er^3+^+Yb^3+^, and HfO_2_: Er^3+^+Yb^3+^+Na^+^ nanoparticles.

Figure 1 shows the XRD diffractograms recorded for HfO_2_ nanoparticles, undoped and codoped with Er^3+^, Yb^3+^, and Na^+^, as obtained by hydrothermal synthesis + TT at 110°. In all the diffractograms of the analyzed samples, the (11) plane at 28° typical of the monoclinic phase was found,^43,48,49^ and the change from the monoclinic to the cubic phase is observed upon addition of the lanthanide ions (Ln^3+^) of Er^3+^ and Yb^3+^ and subsequently upon addition of the Na^+^ cation.

It was reported that the addition of lanthanide ions stabilizes the cubic phase at room temperature, and this was attributed to the presence of oxygen vacancies.^24^ The sites of Hf^4+^ ions are replaced by Ln^3+^ ions in both the monoclinic and cubic lattices.^36,65^ The cubic phase has, as its characteristic, the (111) reflex located at 30°.^3,24,36,65^ Therefore, phase mixing is present after the addition of the dopants into the host lattice, and this behavior is in good agreement with JCPDS No. 00-043-1017^66−70^ for the monoclinic phase and JCPDS No. 01-070-2831^71^ for the cubic phase. In Table 1, the fwhm values and the grain size calculated using the Scherrer formula are listed. For the grain size analysis, the (11) plane was used. There is a decrease in grain size with the addition of Ln^3+^ ions as well as a shift of the peaks, indicating micro strains in the crystalline lattice. The presence of Na^+^ ions induces a deformation in the crystal lattice, causing an increase in grain size, and this behavior was observed with the presence of other alkaline ions such as Li^+^. The increase in the intensity of the (111) peak indicates a stabilization of the cubic phase. The diffractograms corroborate what was seen by Raman, the decrease of the Ag_Hf–O_ mode characteristic of the monoclinic phase suggests a phase mixture.^35^

**Table 1:** Size of HfO_2_, HfO_2_: Er^3+^, Yb^3+^ Nanoparticles

sample	fwhm	crystallite size (nm)
HfO_2_	0.2420	33.8354
HfO_2_: 3 at % Er^3+^/7 at % Yb^3+^	0.2987	27.4104
HfO_2_: 3 at % Er^3+^/7 at % Yb^3+^/20 at % Na^+^	0.2565	31.9150

**Figure 2:**
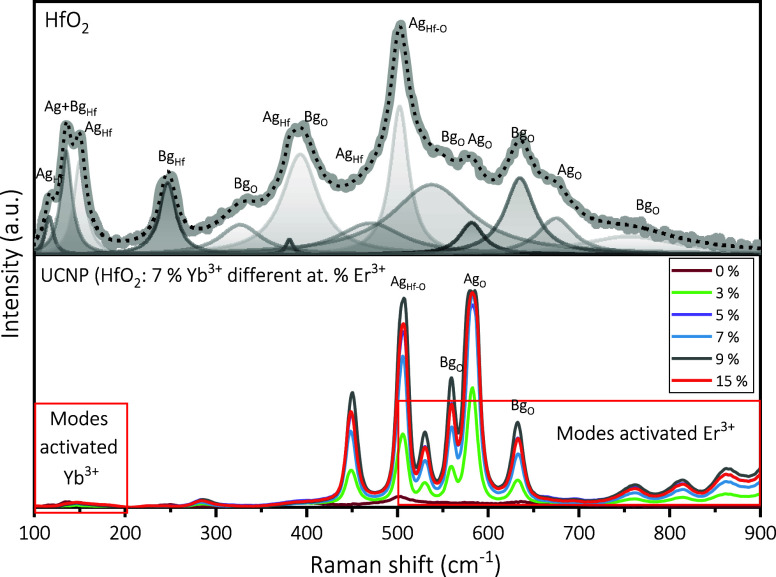
Raman spectra for HfO_2_: 7 at % Yb^3+^ with variation in the Er^3+^ atomic percentage.

Figure 2 shows the Raman spectra of HfO_2_ nanoparticles. The characteristic Ag and Bg modes of HfO_2_ were identified like a previous work, and the most intense peak located at ∼500 cm^–1^ Ag is associated with the Hf–O bond.^41−44,50,65^ The peaks located at lower wavenumbers are mainly due to vibrations of hafnium ions (Hf^4+^), and these peaks are located below ∼300 cm^–1^. The peaks after the most intense peak (∼495 cm^–1^ Ag) are due to exclusive vibrations of oxygen (O^2–^) ions, and this is due to the accommodation of the atoms in a monoclinic cell (Figure 2a). While the modes activated by the dopant ions can be appreciated at Figure 2b, phonons due to Yb^3+^ are located at 100–200 cm^–1^, whereas Er^3+^ vibrational modes have dispersions since 500–1100 cm^–1^.^72^ Therefore, there is a peak superposition between HfO_2_ and Er^3+^ vibrational modes, which could be attributed to a possible substitution of the Hf^4+^ ions by Ln^3+^ ions, which causes changes in the HfO_2_ atomic structure, which consequently generates a change in the vibration of the bonds; the peaks are sharper, and such a behavior has already been reported.^73,74^

The change of the vibrational modes with respect to the doping level is presented in Table 2, and it has been proven that the variation of the doping level does not affect the intensity of the activated Raman modes in an increasing or decreasing way. However, the fwhm of the AgHf-O (∼500 cm^–1^) presents a notorious decrease (77%), which indicates that there is an increase in the crystallinity of the material, and this is characteristic of the doped matrices. A Raman shift of 5 cm^–1^ of this mode is probably due to the stress generated by the Er^3+^ and Yb^3+^ ions inside the host lattice. On the other hand, it has been proved that the variation of the doping level does not affect the intensity of the activated Raman modes in an increasing or decreasing way. However, there is a slight Raman shift of the AgHf-O mode in the undoped HfO_2_ matrix to HfO_2_ codoped samples at 3, 5 y 7% Er^3+^ to 9 y 15% Er^3+^. The shift is due to the stress generated by the Er^3+^ and Yb^3+^ ions inside the host lattice, taking into account that the equipment error is 0.1 cm^–1^. There are reports in which the phase change is mentioned with the addition of ions from monoclinic phase to cubic.^3,23,31,34,35,50^

**Table 2:** Raman Shift Variation and FWHM in Codoped Samples

sample	Raman shift	fwhm
HfO_2_	502.5 cm^–1^	62.0
HfO_2_: 7% Yb^3+^/3, 5, 7% Er^3+^	505.5 cm^–1^	14.5
HfO_2_: 7% Yb^3+^/9, 15% Er^3+^	506.0 cm^–1^	14.0

### Morphological Properties

**Figure 3:**
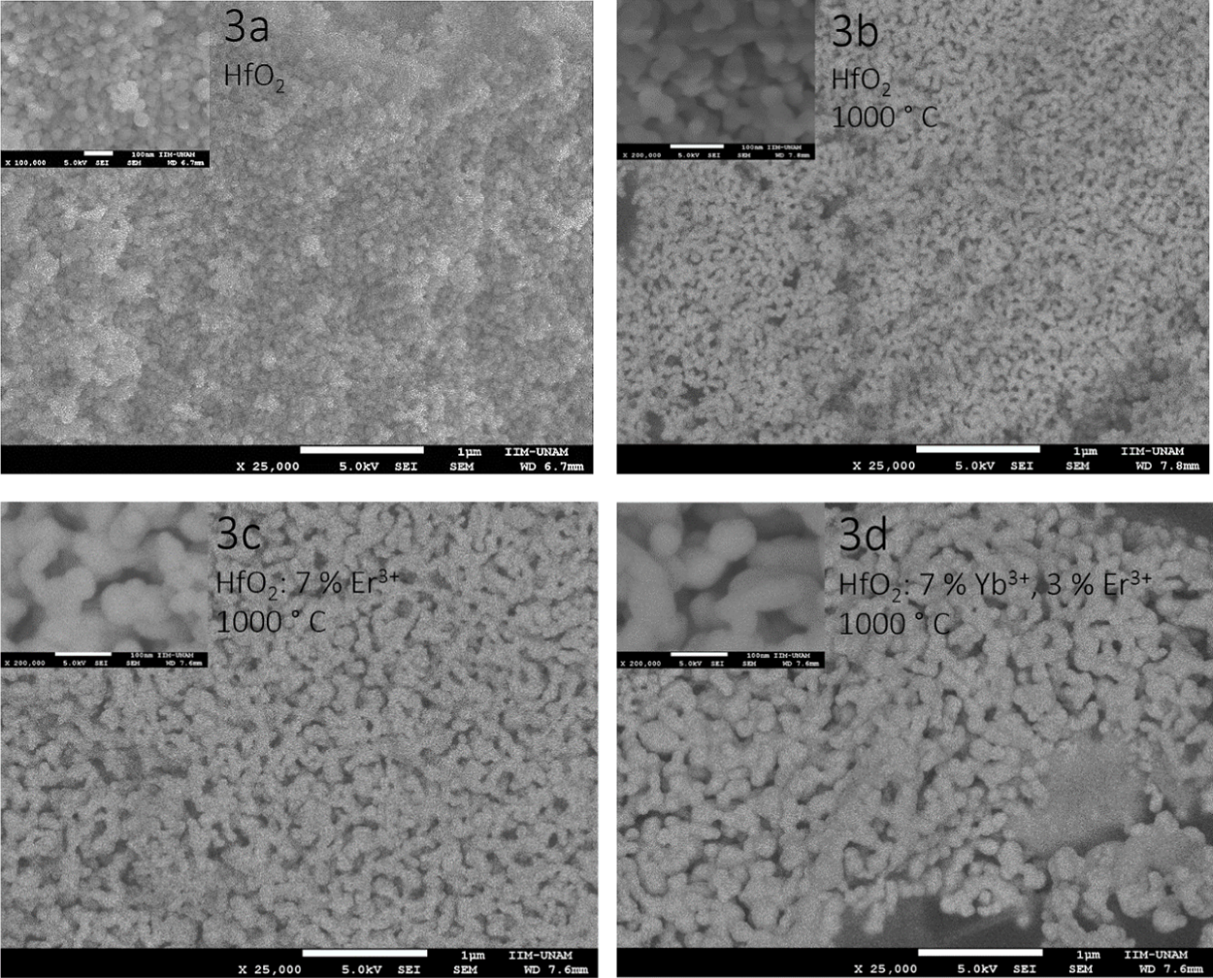
SEM micrographs of undoped and doped HfO_2_ nanoparticles. (a) HfO_2_ without heat treatment, (b) HfO_2_ heat-treated, (c) HfO_2_: Er^3+^ (7 at %) heat-treated, and (d) HfO_2_: Yb^3+^ (7 at %) + Er^3+^ (3 at %) heat-treated.

SEM micrographs of doped and undoped HfO_2_ nanoparticles are displayed in Figure 3. Figure 3a shows the HfO_2_ nanoparticles without heat treatment (at 1000 °C), with a nanoparticle size of ∼60 nm, and their surface is formed of agglomerated spheroidal particles throughout the entire sample. The HfO_2_ nanoparticles which were subjected to a heat treatment of 1000 °C for 2 h are shown in Figure 3b, and in this case, a diameter of ∼80 nm is observed. Here, a small increase in the size of the nanoparticles is found that is probably due to the thermal treatment. In this case, some type of nucleation is observed, changing the surface morphology from a spherical shape to a shape more like an oval or “egg” by the union of the spheroidal nanoparticles.^75−77^ The surface of the HfO_2_: Er^3+^ (7 at %) nanoparticles heat-treated at 1000 °C for 2 h is presented in Figure 3c. The surface looks porous, formed by particles of approximately 90 nm, like the one shown in Figure 3b but larger. Finally, the HfO_2_ nanoparticles doped with 7 at % of Yb^3+^ and 3 at % of Er^3+^ are shown in Figure 3d, and this sample displays agglomerated and porous morphology with particles size of ∼90 to 110 nm. Evidently, the thermal treatment and doping modify the morphology of the samples by promoting the binding of the spheroidal nanoparticles of the initial material. The thermal energy (during the thermal treatment) contributes to the coalescence of the initial particles, increasing their size and their change in shape, and apparently the presence of dopant ions also plays an important role in these changes in the morphology.^34,75−77^ The coalescence effect on particles is common in oxides subjected to high-temperature annealing.^78−80^

**Figure 4:**
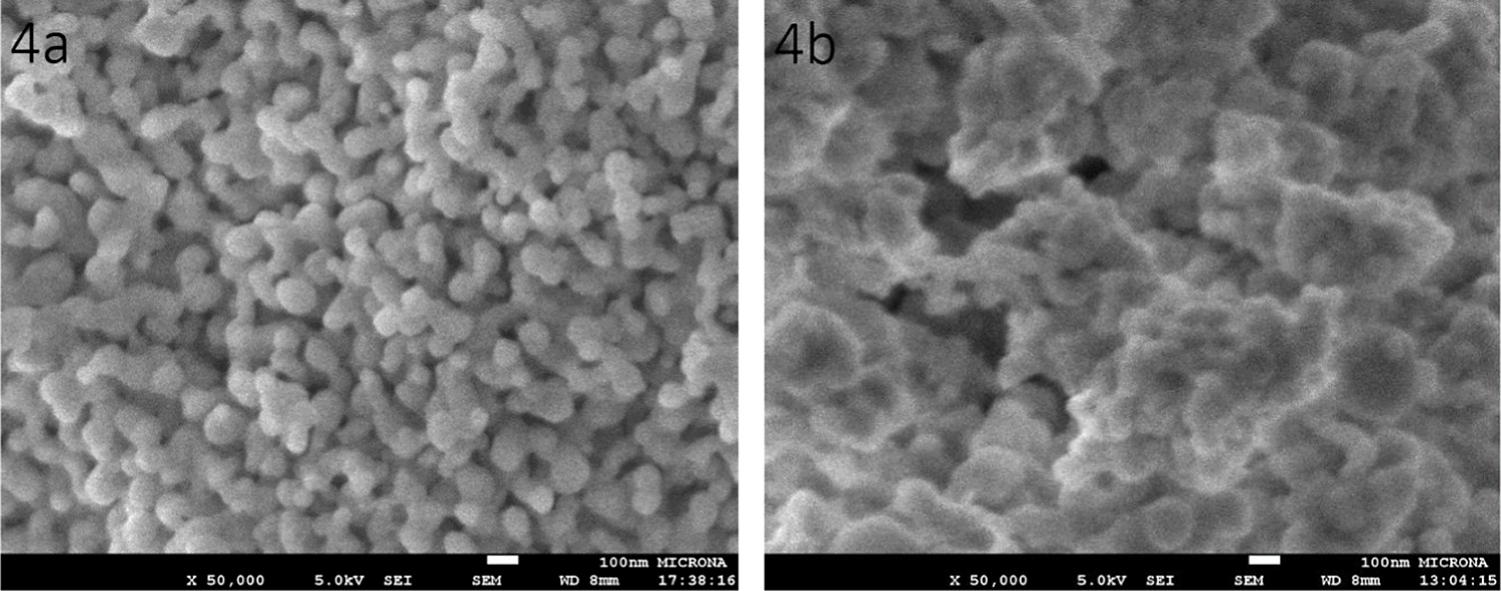
SEM micrographs of codoped HfO_2_ nanoparticles. (a) HfO_2_: Er^3+^ (3 at %) + Yb^3+^ (20 at %) and (b) HfO_2_: Er^3+^ (3 at %) + Yb^3+^ (7 at %) + Na^+^ (20 at %).

Figure 4 shows the SEM micrographs of the codoped samples. The micromorphology of the HfO_2_: 3 at % Er^3+^ + 20 at % Yb^3+^ nanoparticles is shown in Figure 4a. This surface shows agglomerated particles which are shaped like peanuts combined with spheroidal particles with an average size of ∼100 nm and greater. The pattern of HfO_2_: 3 at % Er^3+^ + 7 at % Yb^3+^ + 20 at % Na^+^ is displayed in Figure 4b. The addition of sodium produces a greater nucleation of nanoparticles and a decrease in their homogeneity, presenting particles of 200 nm and more. Evidently, the excessive size of the particles is associated with the presence of sodium ions.

The chemical composition (EDS) results for doped and undoped HfO_2_ nanoparticles are given in Table 3. The undoped HfO_2_ sample shows no traces of impurities, such as chlorine or sodium, despite being precursors of the hydrothermal synthesis, and it is due to the high temperatures of the thermal treatment^75,76^ and to the washing processes during the synthesis. The sample doped nominally with 7 at % Er^3+^ was characterized by the presence of Er, indicating that it was incorporated into the host lattice. According to the results obtained by this technique, it is observed that the stoichiometry of the material is affected by the inclusion of the Er^3+^ ion in comparison with the pure HfO_2_ sample, with a greater variation in the percentage of Hf present in samples doped with 7 at % of Er^3+^. This behavior is similar in the sample codoped with 7 at % Er^3+^ and 3 at % Yb^3+^ with respect to the Yb^3+^ ion.

**Table 3:** Chemical Composition of Undoped, Doped, and Codoped HfO_2_ Nanoparticles

HfO_2_			HfO_2_: 7 at % Er^3+^			HfO_2_: 7 at % Yb^3+^, 3 at % Er^3+^		
element	wt %	at %	element	wt %	at %	element	wt %	at %
O	17.24	69.92	O	16.03	67.21	O	14.54	65.39
Hf	82.76	30.08	Hf	35.15	13.21	Hf	71.37	28.76
			Er	48.82	19.58	Er	0.00	1.00
						Yb	14.08	5.85
totals	100.0		totals	100.0		totals	100.0	

### Photoluminescence Properties

#### Down Conversion

**Figure 5:**
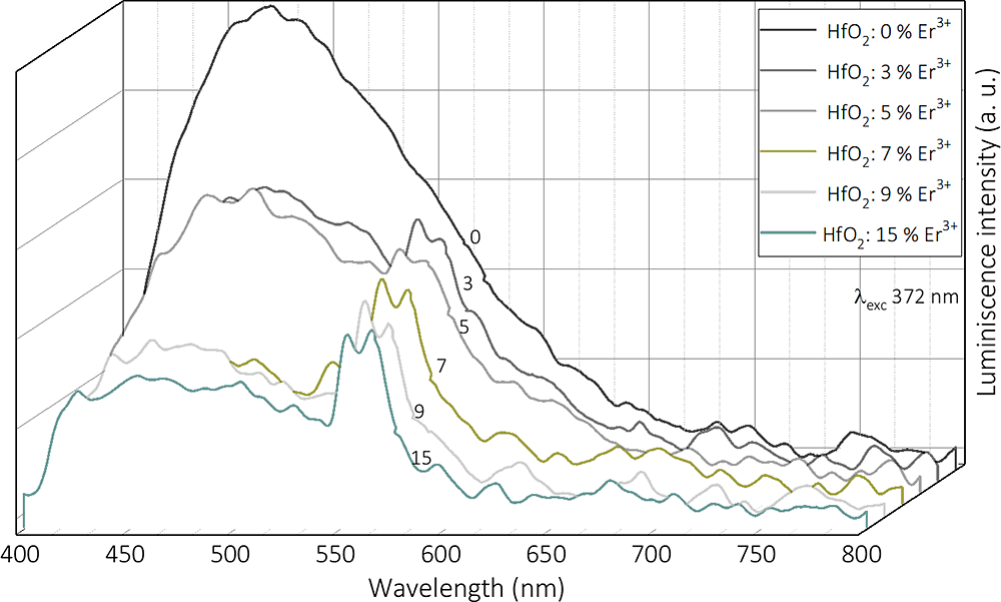
DC photoluminescence of erbium-doped HfO_2_ nanoparticles as a function of the Er^3+^ content. The excitation wavelength is 372 nm.

The PL spectra of DC (down conversion) for HfO_2_:Er^3+^ (0, 3, 5, 7, 9, and 15 at %) nanoparticles under the excitation at 372 nm are presented in Figure 5. In the HfO_2_:Er^3+^ (0 at %) sample, a broad band is noted from 400 to about 600 nm, having a maximum at 475 nm. The band is attributed to V_O4_ and mainly V_O3_ oxygen vacancies, and this is the intrinsic PL emission of the HfO_2_ nanoparticles. As the Er^3+^ content increases, this broad band decreases in intensity, and the typical green bands of Er^3+^ ions appear because with the increase of Er^3+^ ions, the number of defects in the matrix decreases, thus decreasing the cold light characteristic emission of HfO_2_.^81^ The PL emission bands at 530, 553, and 564 nm correspond to the electronic transitions ^2^H_11/2_ → ^4^I_15/2_ and ^4^S_3/2_ → ^4^I_15/2_ of Er^3+^ ions. The red emissions from the Er^3+^ ions are practically negligible. The presence of these bands corroborates that the Er^3+^ ions are embedded in the crystal lattice of the host matrix. It is evident that the maximum PL intensity is reached at 3 at % of Er^3+^ ions, and that in this case, these emissions coexist with the already diminished intrinsic PL emissions from the host lattice. It is also observed that, in the samples doped with 7, 9, and 15 at % of Er ions, the bands in the green region have greater intensity than the broadband from undoped HfO_2_. The rise of doping increases the green PL emission intensity, decreasing, in theory, the number of characteristic oxygen vacancies of HfO_2_. It is known that the trivalent Er^3+^ ions replace the tetravalent Hf^4+^ ions in the crystal lattice, causing the existing vacancies to decrease by the coupling of these ions into the host lattice.^23,81^

#### Up-Conversion

**Figure 6:**
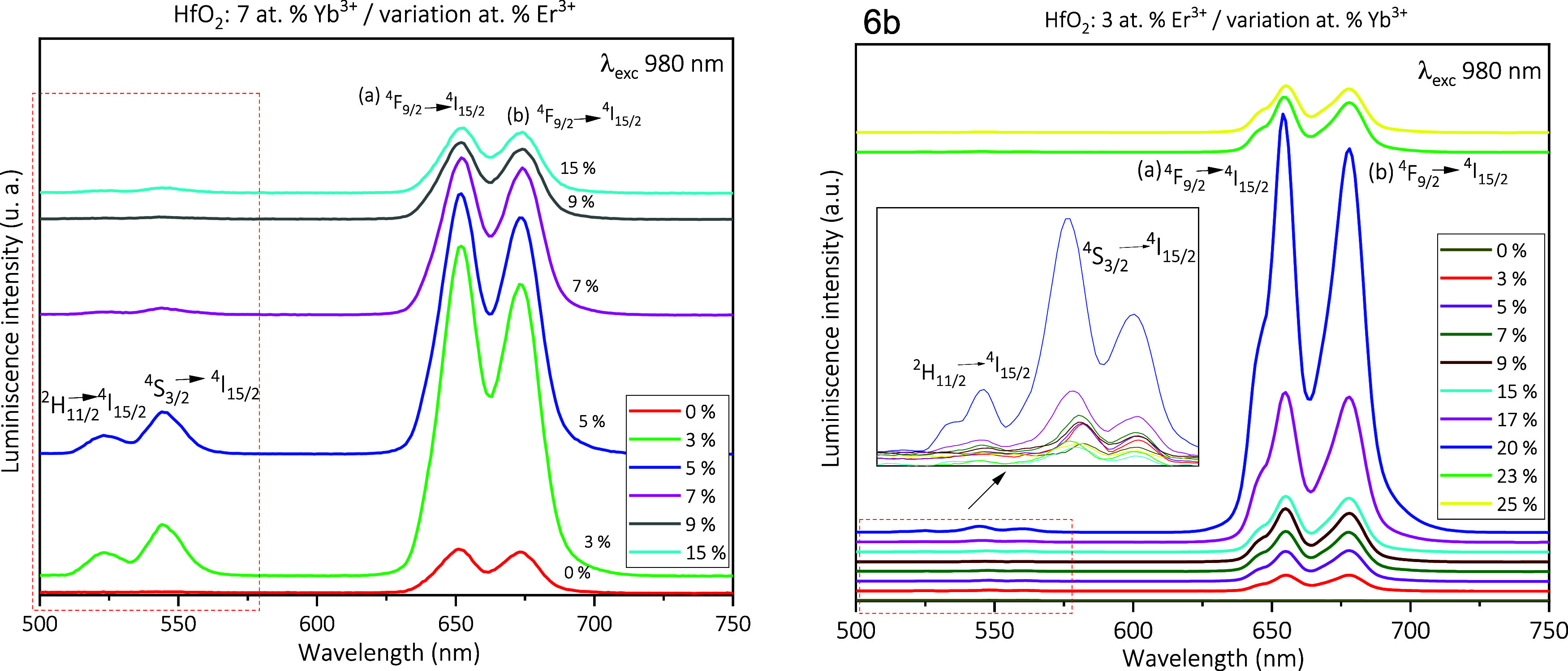
UC luminescence spectra from codoped HfO_2_ nanoparticles under the excitation at 980 nm: (a) Yb^3+^ content is fixed at 7 at %, and Er^3+^ content is varied. (b) Er^3+^ content is fixed at 3 at %, and Yb^3+^ content is varied.

Figure 6a shows the UC (up-conversion) emission spectra for codoped samples: HfO_2_: Yb^3+^ (7 at %) + different atomic concentrations of Er^3+^ ions (0, 1, 3, 5, 7, 9, and 15 at %). The excitation wavelength is 980 nm. The codoping was performed by fixing the Yb^3+^ ion at 7 at %. This was based on other studies performed in HfO_2_^34,40,82,83^ and studies performed with different doping concentrations of Yb^3+^ ions in other host matrices.^7,10,33,51,55,84,85^ In this contribution, it was observed that the increase of Er^3+^ ions raised the UC luminescent intensity, having its maximum threshold at 3 at % of Er^3+^ ions. A decrease of the emission intensity after exceeding this percentage due to the concentration quenching effect was observed. The bands observed at ∼523, 544, 652, and 673 nm correspond to the electronic transitions ^2^H_11/2_ → ^4^I_15/2_, ^4^S_3/2_ → ^4^I_15/2_, and ^4^F_9/2_ → ^4^I_15/2_, respectively, generated by the different energy transfer mechanisms of the up-conversion effect (ESA = excited absorption state, CR = cross relaxation, and ET = energy transfer).^86,87^ Evidently, the UC emissions generated come from the electronic transitions in the Er^3+^ ions.^51,52,55,85^ It was found that varying the amount of Er^3+^ doping in the codoping mostly changes the red emission intensity of Er^3+^ ions, which is of utmost importance for bioimaging applications as it allows to modify the emission intensity because there is a decrease of the UC luminescence in this type of applications.^85^ On the other hand, the red emission allows a greater penetration into the skin, thanks to the infrared excitation which is within the biological window (650–950 nm), which benefits the interaction with the organic tissues.^4^ The inset exhibits an amplification of the green band zone of the UC emission spectrum generated from the Er^3+^ ions. Figure 6b shows the UC emission spectra for codoped samples HfO_2_: Er^3+^ (3 at %) with different atom concentrations of Yb^3+^ ions (0, 3, 5, 7, 9, 15, 17, 20, 23, and 25 at %). The excitation wavelength was 980 nm. Here, the typical emissions from the Er^3+^ ions are observed with greater intensity in the red range, and the green bands are almost imperceptible. The highest UC emission intensity is reached for 20 at % of Yb^3+^ ions; for higher values, a concentration quenching phenomenon is observed.

**Figure 7:**
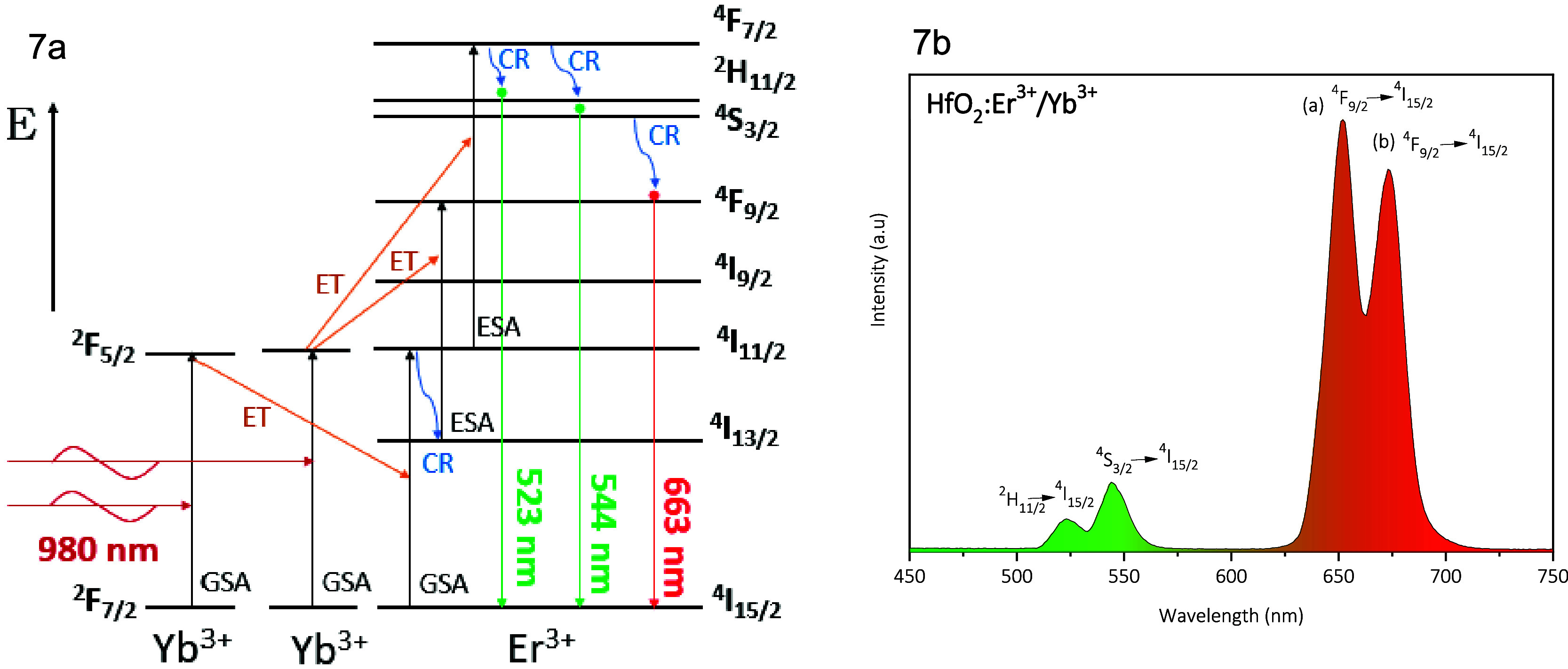
(a) Electronic transitions of the UC process between the Yb^3+^ and Er^3+^ ions and (b) UC emission spectrum of HfO_2_:7 at % Yb^3+^/3 at % Er^3+^ nanoparticles.

Figure 7a,b shows the UC emission bands and possible energy transfer mechanism for HfO_2_:Er^3+^/ Yb^3+^ nanoparticles. This phenomenon begins with the excitation of electrons from the ground state (GSA) ^2^F_7/2_ of the Yb^3+^ ion to the excited state ^2^F_5/2_, followed by the transfer of this energy to an Er^3+^ ion. The bands located at ∼523 and 544 nm, which correspond to the electronic transitions ^2^H_11/2_ → ^4^I_15/2_ and ^4^S_3/2_ → ^4^I_15/2_, respectively, are generated mainly by the energy transfer occurring from Yb^3+^ to Er^3+^. The excitation of the ^4^I_15/2_ ground state of Er^3+^ with the laser energy (980 nm) yields the ^4^I_11/2_ → ^4^I_15/2_ transition, and the energy transfer (ET) from Yb^3+^ (^2^F_5/2_ → ^2^F_7/2_) to Er^3+^ at ^4^I_11/2_ provides the photon summation, which makes the ^2^H_11/2_ → ^4^I_15/2_ and ^4^S_3/2_ → ^4^I_15/2_ transitions possible. While the bands at ∼652 and 673 nm corresponding to the ^4^F_9/2_ → ^4^I_15/2_ transition occur due to the cross relaxation (CR) of radiation existing at higher sublevels (^4^F_3/2_, ^2^H_11/2_, and ^4^S_3/2_), and the excitation of the fundamental state ^4^I_15/2_ of Er^3+^ with the laser energy (980 nm) generating the transition of ^4^I_11/2_ → ^4^I_15/2_ which with the help of the transfer energy from Yb^3+^ to Er^3+^ achieves the transfer of ^4^F_9/2_ → ^4^I_15/2_ so this transition is generated in two emission bands ((a) ∼ 652 and (b) ∼ 673 nm). It is said that for this transition, rigid sublevels are generated, which are caused by the thermal coupling of the ions.^55^

**Figure 8:**
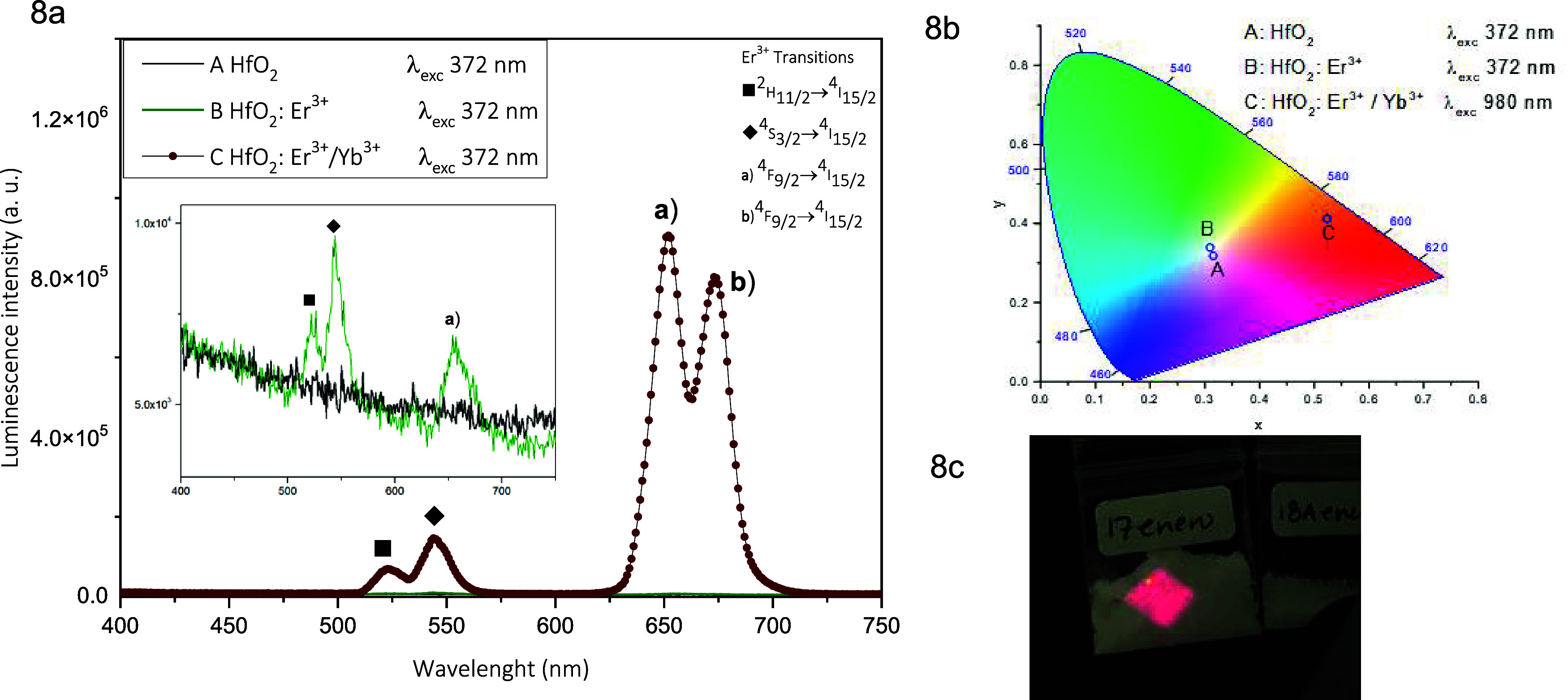
(a) UC emission spectra for HfO_2_ nanoparticles, HfO_2_: 7 at % Er^3+^ and HfO_2_: 7 at % Yb^3+^+ 3 at % Er^3+^ nanoparticles, (b) CIE chromatic coordinates for doped and undoped HfO_2_ nanoparticles, and (c) photographs of UC emissions for HfO_2_: Er (3 at %) + Yb (7 at %) nanoparticles excited with a portable IR laser (980 nm, 0.5 W).

The difference of emission generated in UC in the doped and codoped samples is displayed in Figure 8a. The decrease in the spectrum generated by the oxygen vacancies existing in the HfO_2_ host lattice is observed by adding the Er^3+^ (7 at %) ions, and one can see the up-conversion effect. The Er^3+^ ion is reported as one of the lanthanide ions capable of generating both up-conversion and down conversion emissions because it has appropriate localized states.^3^ In the spectrum of the HfO_2_:Er^3+^ sample, a wavelength of 372 nm was used generating down conversion emissions, and the energy absorption in the sample originates a step in the electrons in the levels ^4^I_11/12_ and ^4^F_7/2_ from where the electron decays in a nonradiative way mainly in the levels ^4^S_3/2_ and ^2^H_11/2_. When the IR radiation (980 nm) absorber (Yb^3+^) is introduced, emissions in the red zone of the Er emission spectrum appear prominently. This comparative study was mainly done to emphasize the large differences in band intensities in the red region when Yb^3+^ ions are introduced. The colors of these emissions are identified with the help of the CIE (Commission International de L'Eclairage) chromatic diagram showing the coordinates (*x*, *y*) for the UC emissions of HfO_2_, HfO_2_: Er (7 at %), and HfO_2_: Er (3 at %) + Yb (7 at %) in Figure 8b. Points A and B are in the white region of the chromatic diagram, although it must be clarified that their emission intensities are low. A very strong intensity is reached in the case of point C located in the red-orange region. Figure 8c shows a photograph taken by ourselves of the up-conversion emission for the HfO_2_: Er (3 at %) + Yb (7 at %) sample excited with a portable IR laser (980 nm, 0.5 W). In this case, the strong intensity of the red-orange UC emission is detected.

**Figure 9:**
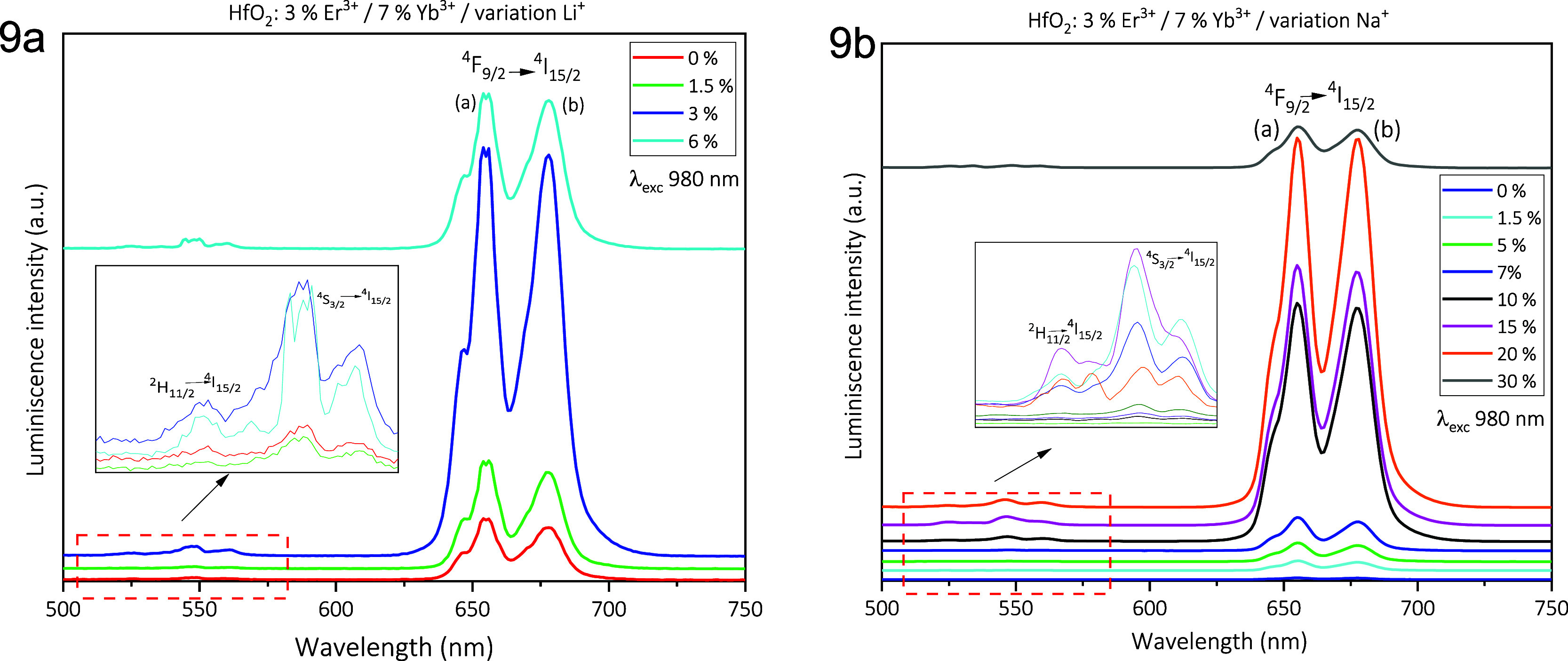
(a) UC emission spectra for HfO_2_: Er^3+^ (3 at %) + Yb^3+^(7 at %) nanoparticles codoped with different lithium concentrations (0, 1.5, 3, and 6 at %) and (b) UC emission spectra for HfO_2_: Er^3+^ (3 at %) + Yb^3+^(7 at %) nanoparticles codoped with different sodium concentrations (0, 1.5, 5, 7, 10, 15, 20, and 30 at %).

The up-conversion emission spectra of the samples containing alkaline ions are shown in Figure 9. It is known in the literature that the incorporation of alkaline cations (Li, Na, K, and so forth) as codopants improves the intensity of UC emission. In Figure 9a, UC emission spectra for HfO_2_: Er^3+^ (3 at %) + Yb^3+^ (7 at %) nanoparticles codoped with different lithium concentrations (0, 1.5, 3, and 6 at %) are exhibited. The excitation wavelength was 980 nm. These UC emission spectra present the distinctive emission bands of the Er^3+^ ions and show different UC emission intensities as the content of Li^+^ ions is increased, and the highest emission intensity is reached with 3 at % Li^+^ ions. The UC emission spectra for HfO_2_: Er^3+^ (3 at %) + Yb^3+^ (7 at %) nanoparticles codoped with different sodium concentrations (0, 1.5, 5, 7, 10, 15, 20, and 30 at %) are displayed in Figure 9b. As in the previous case, the UC emission spectra show the characteristic bands of the Er^3+^ ions with the predominating bands in the red range. Here, it is observed that the maximum emission intensity is reached at 20 at % Na^+^ ions. Presumably, the Li^+^ ions incorporated in the host lattice cause an increase in the emission intensity attributed to the increase of the interatomic distance between the Er^3+^ ions as the Li^+^ ions have a smaller radius with respect to the Hf^4+^ ions. However, further research is necessary to clarify this point.^3,51^

**Figure 10:**
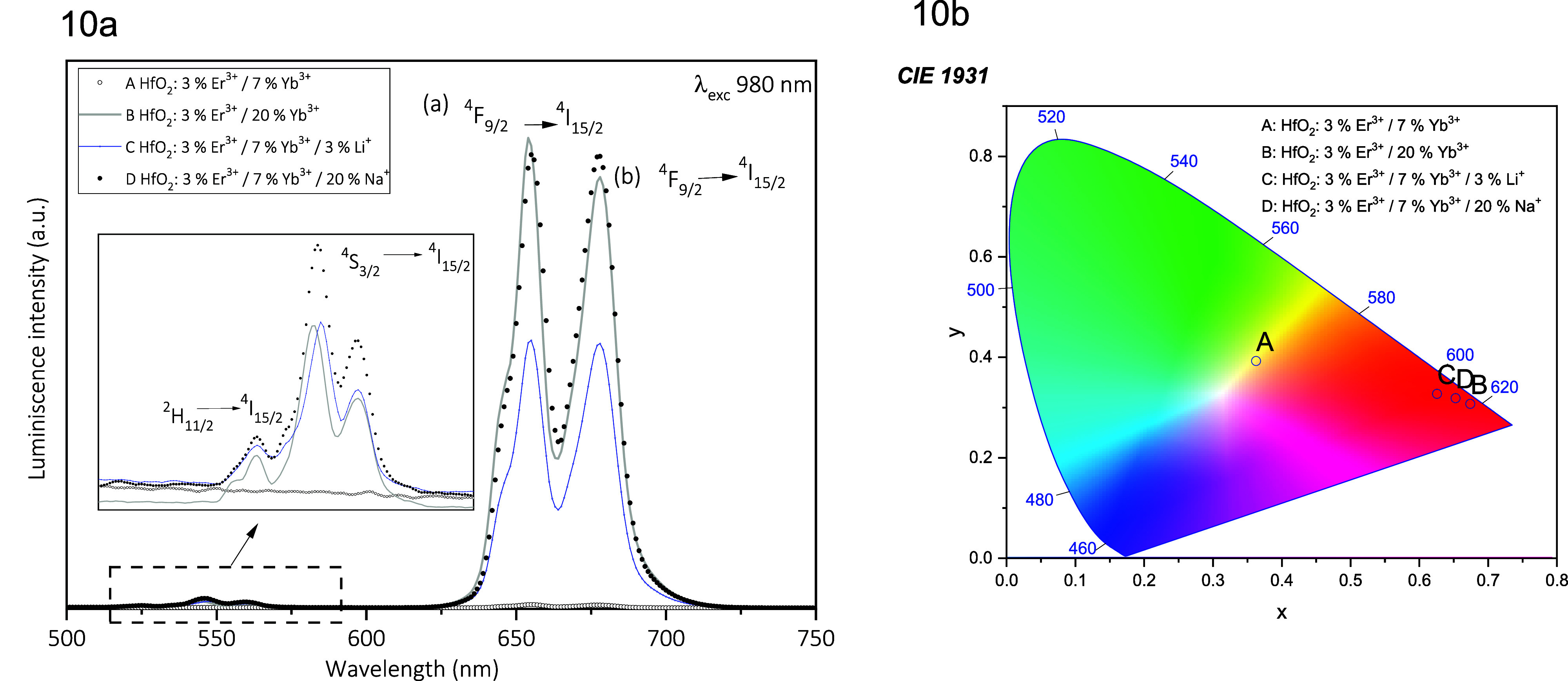
Comparation up-conversion nanoparticles; (a) UC emissions from doped (Er^3+^ + Yb^3+^) and codoped (Li^+^ and Na^+^) HfO_2_ nanoparticles, the excitation wavelength was 980 nm and (b) UC CIE chromatic diagram for.

Figure 10a shows the UC emission spectra for codoped HfO_2_: Er^3+^ + Yb^3+^ with the addition of stabilizer ions, such as Li^+^ and Na^+^ (in all cases, the excitation wavelength was 980 nm). In this figure, curves A and B are for samples without stabilizing ions (Li^+^ and Na^+^). Here, it can be seen that in the case of curve B (with a greater amount of Yb^3+^ ions), its UC emission intensity is much higher than that of curve A. The stabilizing ions (Li^+^ and Na^+^) were incorporated into a sample like the one represented by curve A to better view the effect of their incorporation. It is possible to observe that the incorporation of these ions notably improves the intensity of the red emission coming from the Er^3+^ ions (curves C and D). It is evident that the effect is more notable with the incorporation of Na^+^ ions (curve D). The intensities of curves B and D are very similar. The CIE chromatic diagram for the UC emissions from doped and codoped HfO_2_ nanoparticles is presented in Figure 10b. It should be noted, again, that the emission intensity for the sample represented by point A is very weak compared to that of points B, C, and D, which are much more intense.

### Cathodoluminescence Properties

**Figure 11:**
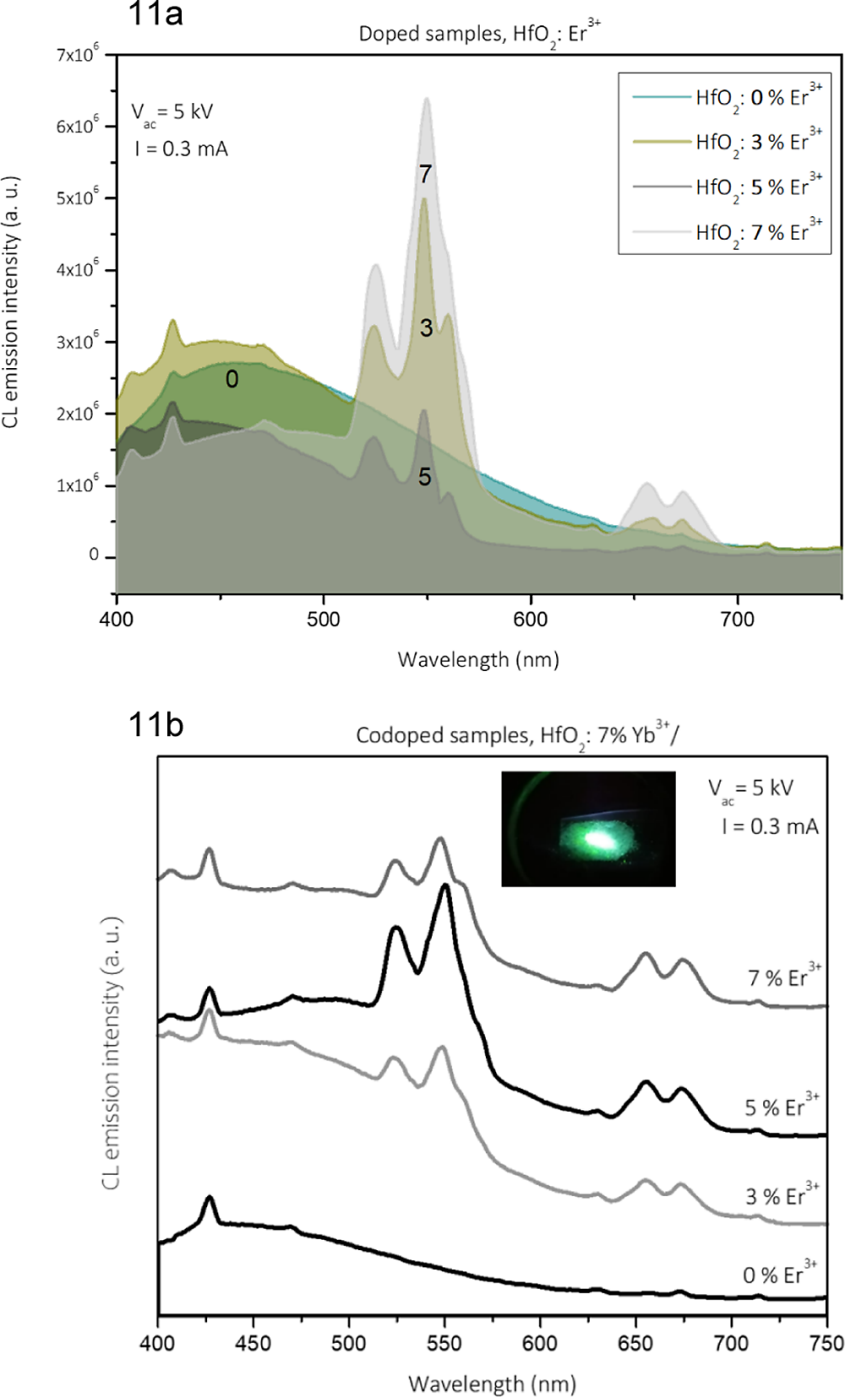
CL emission spectra of (a) HfO_2_ and HfO_2_:Er^3+^nanoparticles as a function of Er^3+^ atomic concentration and (b) HfO_2_:Yb^3+^ (7 at %) + Er^3+^ (X at %) nanoparticles and photograph of green emissions from HfO_2_: 7 Yb^3+^ (at %) + 3 Er^3+^ (at %) nanoparticles excited by accelerated electrons.

Figure 11 shows the cathodoluminescence emission spectra of the Er^3+^-doped HfO_2_ nanoparticles. The spectra of HfO_2_: Er^3+^ (0, 3, 5, and 7 at %) are presented in Figure 11a and those of HfO_2_: (Er^3+^ + Yb^3+^) codoped nanoparticles in Figure 11b. The emission was excited by accelerated electrons (*V* = 5 kV, *I* = 0.3 mA). For HfO_2_: Er^3+^ (0, 3, 5, and 7 at %), the spectrum of the undoped sample exhibits a very broad band that covers almost the entire visible region and shows a maximum around 470 nm. This band is generally associated with structural defects in the host lattice such as oxygen vacancies. As the Er^3+^ ions are incorporated, sharp bands appear in the band, which are typical of this ion.^87^ It should be noted that the bands in the green region are more intense than those in the red region. The emission intensity reaches its maximum for 7 at % Er^3+^ ions. In Figure 11b, the HfO_2_: Yb^3+^ (7 at %) + Er^3+^ (0, 3, 5, and 7 at %) samples were studied. The sample without Er^3+^ ions presents a very broad band without characteristic sharp peaks. As the Er^3+^ ions were incorporated, their typical bands appeared, with those in the green range being more intense. The maximum intensity is reached at 5 at % Er^3+^ ions; for higher contents of Er^3+^ ions, a decrease in the CL emission intensity is seen, probably due to the concentration quenching effect. Here, an intense green emission is observed, and the central spot that looks white is due to the high intensity of the emitted light.

### Significant Results

HfO_2_ nanoparticles codoped by Yb^3+^ (7 at %) + Er^3+^ synthesized by hydrothermal and thermal treatment at 110 °C have the particle size (less than 100 nm) optimum to be used for interaction with cells; others morphology characteristics as monodispersion are considered. HfO_2_ possesses oxygen vacancies that play an important role in the cooping process. UCNP has a great red-orange emission, at room environment. Red emission was improved by the study of different compositions and active cations like Na^+^ and K^+^; additionally, UCNP nanoparticles possess an exponential red-orange emission increase compared with green emission. Therefore, all these properties suggest that our UCNP could be applied as biomarkers or in phototherapy.

## Conclusions

Using the simple and economical hydrothermal technique, doped and undoped HfO_2_ nanoparticles were synthesized. The crystalline structure of these nanoparticles is mostly monoclinic in the undoped samples and evolves to a mixture of the monoclinic and cubic phases when dopant ions are introduced. Regarding the structural properties, the decrease in the Ag_Hf–O_ mode observed in Raman suggests phase mixing of monoclinic and cubic phases, which was corroborated by XRD results. With the addition of trivalent ions, stabilization of the cubic phase was obtained at low temperatures, and a higher stabilization of the cubic phase was observed with the addition of Na^+^ ions. The EDS measurements of the chemical composition of the nanoparticles confirm correct stoichiometry. The surface morphology of HfO_2_ nanoparticles was characterized by the presence of spheroidal particles and pores before thermal treatment, and it changed to larger peanut-shaped particles after the thermal treatment. These nanoparticles have notable luminescence characteristics. On the one hand, they show luminescence emissions when only doped with Er^3+^ ions and excited with ultraviolet radiation (272 nm), and in this case, emissions in the green range of the spectrum prevail. On the other hand, when they were doped with Er^3+^ and Yb^3+^ ions, strong red emission was obtained under the excitation with infrared radiation (980 nm). The UC emission intensity of these samples was notably increased with the help of stabilizers such as Li^+^ and Na^+^, and this effect was stronger for Na^+^ ions. Cathodoluminescence measurements show strong emissions preferably in the green region of the spectrum. Variations in the atomic concentrations of the dopant ions allowed us to determine the optimal values of each of them and to know the thresholds at which the concentration quenching effect occurs.

Finally, HfO_2_ nanoparticles doped and codoped with Er, Yb, Li, and Na ions show very versatile and interesting luminescence properties since they allow obtaining strong emissions excited either with ultraviolet, infrared radiation, or accelerated electrons. These characteristics allow a variety of applications of these materials; particularly, they are excellent candidates to be utilized in medical applications, specifically, for cancer biomarker applications.
